# Memristor Crossbar Circuits Implementing Equilibrium Propagation for On-Device Learning

**DOI:** 10.3390/mi14071367

**Published:** 2023-07-03

**Authors:** Seokjin Oh, Jiyong An, Seungmyeong Cho, Rina Yoon, Kyeong-Sik Min

**Affiliations:** School of Electrical Engineering, Kookmin University, Seoul 02707, Republic of Korea; ghj163@kookmin.ac.kr (S.O.); sunday1903@kookmin.ac.kr (J.A.); jo1056@kookmin.ac.kr (S.C.); flsk0419@kookmin.ac.kr (R.Y.)

**Keywords:** memristor crossbar circuits, equilibrium propagation, on-device learning, local learning

## Abstract

Equilibrium propagation (EP) has been proposed recently as a new neural network training algorithm based on a local learning concept, where only local information is used to calculate the weight update of the neural network. Despite the advantages of local learning, numerical iteration for solving the EP dynamic equations makes the EP algorithm less practical for realizing edge intelligence hardware. Some analog circuits have been suggested to solve the EP dynamic equations physically, not numerically, using the original EP algorithm. However, there are still a few problems in terms of circuit implementation: for example, the need for storing the free-phase solution and the lack of essential peripheral circuits for calculating and updating synaptic weights. Therefore, in this paper, a new analog circuit technique is proposed to realize the EP algorithm in practical and implementable hardware. This work has two major contributions in achieving this objective. First, the free-phase and nudge-phase solutions are calculated by the proposed analog circuits simultaneously, not at different times. With this process, analog voltage memories or digital memories with converting circuits between digital and analog domains for storing the free-phase solution temporarily can be eliminated in the proposed EP circuit. Second, a simple EP learning rule relying on a fixed amount of conductance change per programming pulse is newly proposed and implemented in peripheral circuits. The modified EP learning rule can make the weight update circuit practical and implementable without requiring the use of a complicated program verification scheme. The proposed memristor conductance update circuit is simulated and verified for training synaptic weights on memristor crossbars. The simulation results showed that the proposed EP circuit could be used for realizing on-device learning in edge intelligence hardware.

## 1. Introduction

Recently, deep neural networks (DNNs) have exhibited remarkable performance improvements in applications such as image classification and natural language processing [1,2,3,4,5]. Typically, DNNs are trained offline using backpropagation-based learning algorithms. The backpropagation learning algorithm computes synaptic weights to be updated according to the gradient descent from both forward and backward paths [6,7,8]. However, implementing DNN learning using backpropagation requires complex digital circuits that are unsuitable for edge intelligence hardware, which requires simple circuits with low power consumption [9,10,11,12,13,14,15]. Furthermore, backpropagation is a nonlocal learning algorithm that requires a significant amount of buffer memory to store all the neuronal and synaptic information from an entire network [16,17,18,19,20]. Alternatively, brain-mimicking learning algorithms, such as spike-timing-dependent plasticity (STDP), can be considered [21,22,23,24,25]. Although STDP requires much simpler hardware than backpropagation, the performance of DNNs trained by STDP is still unsatisfactory compared to backpropagation learning [24,26,27].

Equilibrium propagation (EP) has been proposed as a new neural network learning algorithm based on a local learning concept, where only local information is used to calculate the synaptic weight update [28,29,30,31]. In contrast to global learning, in local learning, each synaptic weight is updated using only local information, such as the neighbor’s neuronal activations. Because of the local learning nature of EP, the need for a very large buffer memory for storing the information of the entire network can be eliminated in the EP hardware, which makes it very suitable for edge intelligence hardware.

However, one issue with the EP algorithm is that the EP neural dynamics should be solved numerically, not analytically. Numerical iteration to solve the EP dynamics can make the computation inefficient in terms of calculation accuracy and computing energy compared to backpropagation, which can calculate the weight update analytically. Digital computers require long computation time and a large amount of computing energy when performing numerical iteration to solve the EP dynamics. Instead of numerical iteration on a digital computer, analog circuits can be considered for solving the EP neural dynamics physically, not numerically [32]. In the previous work, the EP dynamic equations were solved with Kirchhoff’s current and voltage laws using analog circuits [32,33]. This method considerably reduces the calculation time because there is no numerical iteration [32].

Despite the improvement due to physics-based equation solving, the existing analog circuit technique has a significant problem. The free-phase solution should be stored in some analog voltage memories, such as very large capacitors, because it needs to be used later together with the nudge-phase solution for calculating the synaptic weight update [32]. Alternatively, if analog voltage memories are not used, digital memories with analog-to-digital and digital-to-analog conversion circuits should be incorporated into the physics-based equation-solving circuits for storing the free-phase solution temporally. In this case, the added circuits, such as the conversion and memory circuits, can increase the layout area and consume more power. Therefore, to make the EP circuit useful for edge intelligence hardware, the need for storing the free-phase solution should be avoided. Moreover, one more important thing to note is that some essential peripheral circuits, such as the neuron circuit and weight update circuit, were lacking in previous work. The lack of peripheral circuits prevents the practical utilization of an EP circuit in edge intelligence hardware.

Therefore, a new analog circuit technique to realize the EP algorithm in practical and implementable hardware is proposed herein. To implement this technique, this work makes two contributions. First, the free-phase and nudge-phase solutions are calculated by the proposed analog circuits simultaneously instead of at different times. Accordingly, the analog voltage memories or digital memories with converting circuits between digital and analog domains for storing the free-phase solution temporarily can be eliminated in the proposed EP circuit. Second, a simple EP learning rule relying on a fixed amount of conductance change per programming pulse is proposed and applied in peripheral circuits. The modified EP learning rule makes the weight update circuit practical and implementable without the use of complicated program verification schemes. The memristor conductance update circuit proposed herein was simulated and verified for training synaptic weights on memristor crossbars. The simulation results indicated that the proposed EP circuit is suitable for edge intelligence hardware for realizing physics-based on-device learning.

## 2. Method

### 2.1. Algorithm

The EP algorithm is a learning framework for energy-based models. Its energy can be defined as a type of Hopfield energy, E, using the following equation [34]:

(1) $$Energy=\frac{1}{2}\sum_{i}u_{i}^{2}-\frac{1}{2}\sum_{i\neq j}W_{ij}\rho (u_{i})\rho (u_{j})$$

where *u* denotes the state of a neuron, *W* is a synaptic weight between two neurons, *ρ* is the nonlinear activation function, and *i* and *j* represent neuron numbers.

**Figure 1:**
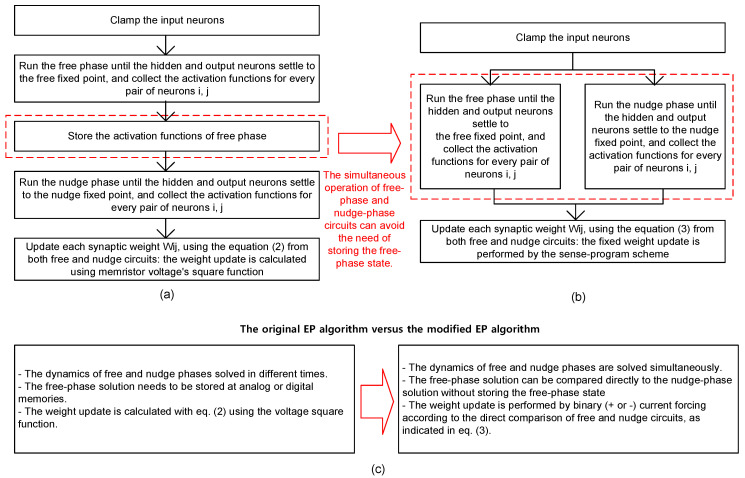
(**a**) Flowchart of the original EP algorithm that solves the free-phase and nudge-phase dynamics in different times; (**b**) flowchart of the proposed EP algorithm modified for solving the free-phase and nudge-phase dynamics simultaneously; (**c**) summary of the differences between the original and modified EP algorithms.

The EP algorithm modeled with the Hopfield energy can be considered an alternative way to the conventional backpropagation algorithm, in which very complicated digital circuits external to neural network neurons and synapses are needed for performing its global learning process [28]. Unlike the backpropagation algorithm, the EP algorithm with Hopfield energy can be trained using the local learning rule, where a weight update can be calculated using information from only neighboring neurons. The hardware for local learning can be implemented using much simpler analog circuits, which can be regarded as being more similar to the real operation of the human brain [21,22,23,32]. Figure 1a presents a flowchart of the original EP algorithm [28]. As indicated in Figure 1a, the operation of EP comprises two phases: free and nudge phases [28]. The dynamic equations of the free and nudge phases in Figure 1a are solved one by one at different times.

First, consider the free phase in the original EP algorithm shown in Figure 1a. To calculate the free-phase solution, the input neurons are clamped by the input data. Then, the output neurons should be disconnected from the target labels to keep the neural network unaffected by the target labels. During the free phase, the neurons can be free from the target labels. In particular, the neural network is driven toward an equilibrium state step by step according to the local learning rule of the EP during the free phase. In this step, each neuron updates its activation according to the activity of its neighboring neurons. This local learning step is repeated until the network reaches a stable state. After reaching the stable point, the neuronal activations should be stored in analog or digital memories for later use in calculating the weight updates.

As shown in Figure 1a, the nudge phase follows the free phase. At the following nudge phase, the difference between the output neuron and the target label is computed, and the error is used to drive the network toward a new equilibrium state of the nudge phase. To find the nudge-phase solution, another round of local learning should be performed. Like the free phase, this local learning step is repeated until the network converges to a new stable state during the nudge phase. After reaching the stable state, the weight updates can be calculated using the neural activations collected from both the free and nudge phases. One thing to note here is that the free-phase neuronal activations should be stored in memories for later use in calculating the weight updates after the nudge-phase calculation is finished. To store the neuronal activations from the free phase, analog or digital memory with converting circuits between analog and digital domains should be incorporated into the EP hardware, as explained earlier.

Figure 1b shows a flowchart of the proposed EP algorithm, which is modified for solving the free-phase and nudge-phase dynamics simultaneously. In Figure 1b, because the free and nudge phases are run simultaneously, the free-phase solution can be compared directly with the nudge-phase solution to calculate the synaptic weight update. Because of this procedure, analog voltage memories or digital memories with converting circuits between analog and digital domains are not needed in the EP hardware implementation.

Figure 1c summarizes the difference between the original EP algorithm shown in Figure 1a and the modified EP algorithm shown in Figure 1b, showing that the dynamic equations of the free and nudge phases are solved at different times in the original EP algorithm. Thus, the free-phase solution should be stored in analog or digital memories with converting circuits between analog and digital domains for calculating the weight update later using both free-phase and nudge-phase solutions. Unlike the original EP algorithm, the free-phase and nudge-phase solutions are obtained simultaneously in the modified EP algorithm. Therefore, analog or digital memory can be avoided in the hardware implementation of the modified EP algorithm shown in Figure 1b.

Furthermore, Figure 1c shows that the weight update calculation of the modified EP algorithm is much simpler than the original EP. The original EP learning algorithm calculates memristor conductance updates according to the following equation [28,29,35].

(2) $${∆g}_{ij}\propto -\left[{\left(∆V_{ij}^{\beta }\right)}^{2}-{\left(∆V_{ij}^{0}\right)}^{2}\right]$$

where ${∆g}_{ij}$ represents the amount of memristor conductance update between node *i* and node *j*. $∆V_{ij}^{\beta }$ is the memristor voltage between node *i* and node *j* for solving the nudge-phase equations physically. For calculating the nudge-phase solution, the training target labels should be applied to the output neurons. $∆V_{ij}^{0}$ is the memristor voltage for solving the free-phase equations physically when the output neurons are disconnected from the target labels. In terms of circuit implementation, implementing analog circuits that can calculate the weight update ${∆g}_{ij}$ according to Equation (2) appears very difficult. Specifically, Equation (2) requires an analog voltage multiplier for calculating the voltage square function. Moreover, the weight update ${∆g}_{ij}$ is an analog value depending on the gap between the squares of the free and nudge voltages. Programming the memristor conductance according to ${∆g}_{ij}$ requires very complicated programming and verifying circuits, such as incremental step pulse programming (ISPP) circuits [36,37].

To avoid the need of an analog voltage multiplier and complicated programming and verifying circuits, the weight update equation was modified to be much simpler in this work:

(3) $${∆g}_{ij}=-sgn\left[\left|∆V_{ij}^{\beta }\right|-\left|∆V_{ij}^{0}\right|\right]\times ∆g_{fixed}$$

where sgn denotes a function that outputs the sign of $\left|∆V_{ij}^{\beta }\right|-\left|∆V_{ij}^{0}\right|$. $∆g_{fixed}$ represents a fixed amount of memristor conductance change. In terms of circuit implementation, implementing the circuit version of Equation (3) can be much simpler than that of Equation (2). This is because Equation (3) does not need any analog voltage multiplier. Unlike Equation (2), the voltage square function is not used in Equation (3). Instead of the square function, only the magnitude of the memristor voltage is compared directly between the free and nudge phases to increase or reduce the memristor conductance by a fixed change as small as $∆g_{fixed}$. Updating of the memristor conductance by $∆g_{fixed}$ can be implemented using a very simple memristor conductance programming circuit instead of the very complicated ISPP scheme.

### 2.2. Circuit Implementation

**Figure 2:**
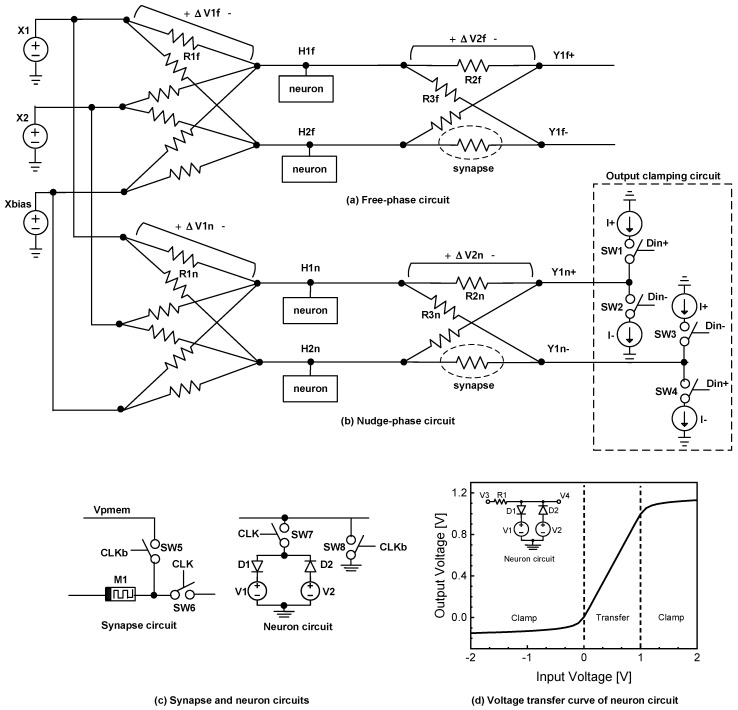
(**a**) Memristor–CMOS hybrid circuit for calculating the free-phase solution that is not affected by the training vectors; (**b**) memristor–CMOS hybrid circuit for calculating the nudge-phase solution with the output clamping circuit; (**c**) synapse and neuron circuits; (**d**) voltage transfer curve of the neuron circuit. Here, the upper and lower limit voltages can be controlled by V1 and V2, respectively.

In this section, the implementation of the modified EP algorithm shown in Figure 1b using analog circuits is described. As explained, the EP equations should be solved physically by simultaneous operation of the free-phase and nudge-phase circuits. Figure 2a depicts a memristor–CMOS hybrid circuit for solving the free-phase equations physically. In Figure 2a, the output nodes are not connected by the output clamping circuit. Accordingly, the free-phase circuit in Figure 2a can calculate the free-phase solution that is not affected by the training vectors. By contrast, Figure 2b shows a memristor–CMOS hybrid circuit for obtaining the nudge-phase solution, where the output neurons are clamped by the training vectors. Therefore, the nudge-phase solution can reflect the training vectors totally, while the free-phase solution is not related to the training vectors. By repeating the weight update continually according to the EP training rule given by Equation (3), the error between the free-phase circuit prediction and the target becomes smaller, as will be explained in the following Results section.

To explain the simultaneous operation of free-phase and nudge-phase circuits in detail, consider Figure 2a–c. Figure 2a,b shows the free-phase and nudge-phase circuits, respectively. Figure 2c shows neuron and synapse circuits, which are common to both the free-phase and nudge-phase circuits. Here, X1, X2, etc., represent input voltages, which clamp the input neurons shared in both free-phase and nudge-phase circuits. H1f, H2f, etc., are node voltages of hidden neurons in the free-phase circuit in Figure 2a. H1n, H2n, etc., are node voltages of hidden neurons in the nudge-phase circuit in Figure 2b. Y1f+ and Y1f- are positive and negative voltages of output neurons in the free-phase circuit, respectively. Similarly, Y1n+ and Y1n- are positive and negative voltages from output neurons in the nudge phase, respectively. In Figure 2a, R1f, R2f, etc., are memristors in the free-phase circuit. In Figure 2b, R1n, R2n, etc., are memristors in the nudge-phase circuit. The memristor conductance represents a synaptic weight between two neurons in Figure 2a,b. For example, the conductance of R1f means a synaptic weight between the input neuron X1 and the hidden neuron H1f in the free-phase circuit. Similarly, the conductance of R1n denotes a weight between X1 and H1n in the nudge phase. ∆V1f and ∆V2f are memristor voltages across R1f and R2f, respectively, in the free-phase circuit. ∆V1n and ∆V2n are memristor voltages across R1n and R2n, respectively, in the nudge-phase circuit.

One difference between the free-phase and nudge-phase circuits is that the output clamping circuit can be found only in the nudge-phase circuit, as shown in Figure 2b. The output clamping circuit is composed of switches and current sources. The switches are controlled by the training vectors Din+ and Din-. If Din+ and Din- are high and low, respectively, SW1 and SW4 are turned on. At the same time, SW2 and SW3 are turned off. Accordingly, Y1n+ and Y1n- are driven by I+ and I-, respectively. When Din+ and Din- are low and high, respectively, Y1n and Y1n- are forced inversely from the previous case. I+ and I- represent a positive and negative current source, respectively, which have the same magnitude but opposite signs. Another notable point is that the magnitudes of the current sources, I+ and I-, are fixed by one value regardless of the amount of output error between the prediction and the target. The fixed magnitudes of I+ and I- are much simpler for implementation with an analog circuit than the variable current sources. Owing to the output clamp circuit in Figure 2b, only the nudge-phase circuit is affected by the training vectors. Further, as the training continues according to the modified EP learning rule of Equation (3), the output of the free-phase circuit becomes more similar to the target.

**Figure 3:**
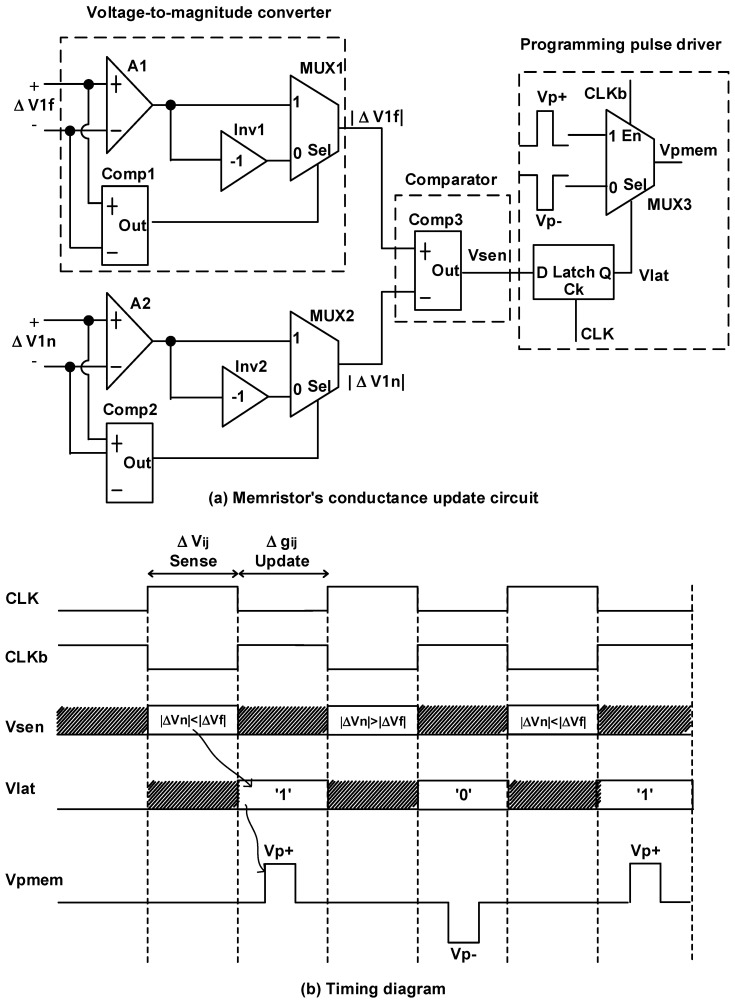
(**a**) Memristor conductance update circuit. (**b**) Timing diagram of the memristor conductance update circuit with magnitude comparison and programming pulse driving.

Figure 2c indicates the neuron and synapse circuits used in Figure 2a,b. In the synapse circuit in Figure 2c, M1 represents a synaptic memristor. When CLK is high, SW6 is on and SW5 is off. At this time, M1 is connected to the neurons. When CLK is low, SW5 is on and SW6 is off, applying a programming voltage pulse of Vpmem to M1. Vpmem is delivered from the memristor conductance update circuit, which is illustrated in Figure 3a. For the neuron circuit in Figure 2c, when CLK is high, SW7 is on and SW8 is off. D1 and D2 are connected to V1 and V2, respectively. Actually, D1 and D2 can limit the voltage transfer curve like the rectified linear unit (ReLU) function. Here, the limit voltages can be controlled by V1 and V2. When CLK is low, SW7 is off and SW8 is on. In that case, the neuron node is connected to the ground voltage, and the activation function of the neuron circuit is disabled.

The voltage transfer function of the neuron circuit is shown in Figure 2d. Here, the simulated neuron circuit is shown in the inset. V1 and V2 are the voltage sources for controlling the upper and lower limit voltages, as explained in Figure 2c. D1 and D2 are diodes connected to V1 and V2, respectively. V3 is an input voltage applied to the neuron circuit through R1. V4 is an output voltage of the neuron circuit. If an input voltage V3 is applied through R1, an output voltage V4 can be found, as shown in Figure 2d. As explained earlier, V1 and V2 can control the upper and lower limit voltages, respectively. D1 and D2 are antiparallel diodes connected to V1 and V2, respectively. The voltage transfer curve is shown in Figure 2d. When V3 is lower than 0 V, D2 is turned on, which causes V4 to be limited by approximately −0.1 V. On the contrary, when V3 is higher than 1 V, D1 is turned on. At this moment, V2 is limited by around 1 V. Thus, the upper and lower limit voltages are approximately −0.1 and +1 V, respectively, which are controlled by V1 and V2, as shown in Figure 2d. The voltage transfer curve in Figure 2d appears very similar to the ReLU function.

Figure 3a shows a memristor conductance update circuit that compares the memristor voltage magnitude between the free-phase and nudge-phase circuits and drives the programming pulse to the memristor synapse shown in Figure 2c according to the comparison result. Here, ∆V1f and ∆V1n are from the free-phase and nudge-phase circuits shown in Figure 2a,b, respectively. The circuit is composed of a voltage-to-magnitude converter, comparator, and programming pulse driver, as depicted in Figure 3a.

In the voltage-to-magnitude converter, A1 and Inv1 are the voltage buffer and voltage inverter, respectively. The operation of the voltage-to-magnitude converter is as follows. First, ∆V1f enters Comp1, which detects the sign of ∆V1f. If the sign of ∆V1f is positive, Comp1 generates 1. Consequently, MUX1 delivers the output of the voltage buffer to the following stage. If the sign of ∆V1f is negative, Comp1 generates 0. At this time, MUX1 delivers the inversion of ∆V1f to the following stage instead of the voltage buffer output. Accordingly, MUX1 delivers the magnitude of ∆V1f to the following Comp3 regardless of the sign of ∆V1f. Similarly, MUX2 delivers the magnitude of ∆V1n to Comp3 regardless of the sign of ∆V1n.

The comparator Comp3 decides which of the two magnitudes of ∆V1f and ∆V1n is larger. The comparator output is connected to the programming pulse driver. In particular, Vsen enters a D latch. The D latch is made of the gated data latch, where the gate signal is CLK and the latch input is Vsen. Further, the latch output is the Vlat signal. The MUX is controlled by the latch output Vlat, as shown in Figure 3a. Moreover, MUX3 is enabled only when CLKb is high. MUX3 has two inputs, Vp+ and Vp-, which are positive and negative programming pulses for increasing and decreasing the memristor conductance, respectively. The MUX output is Vpmem, which is used as a programming voltage pulse for updating the memristor conductance.

Figure 3b shows a timing diagram of the memristor conductance update circuit composed of the voltage-to-magnitude converter, magnitude comparator, and programming pulse driver, as shown in Figure 3a. In Figure 3b, CLK is a reference clock signal. CLKb is an inversion of the CLK signal. Actually, when CLK is high and CLKb is low, the free-phase and nudge-phase circuits are run simultaneously. Accordingly, the memristor conductance update circuit can sense and compare the magnitude of the memristor voltage between the free and nudge phases, as shown in Figure 3a. By contrast, when CLK is low and CLKb is high, the comparator output Vsen is maintained at the latch. During this time, the latch output Vlat can control MUX3 in Figure 3a. Consequently, MUX3 can deliver Vp+ or Vp- to increase or reduce the memristor conductance according to the magnitude comparison of memristor voltage between the free and nudge phases. Vp and Vn are the positive and negative programming pulses, respectively, as indicated in Figure 3a. Vpmem is the output of MUX3 gate in Figure 3a.

## 3. Results

**Figure 4:**
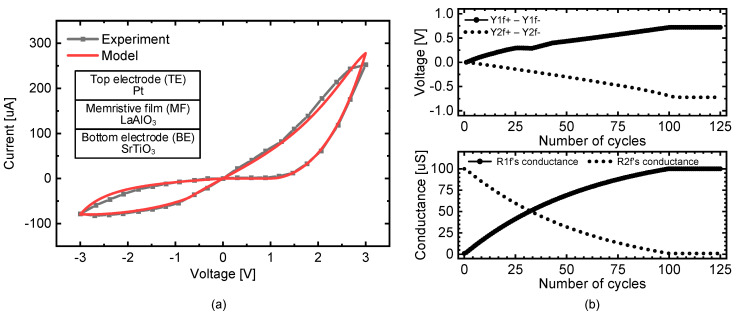
(**a**) Cross-sectional view of the fabricated memristor and its butterfly curve [36]. (**b**) Simulated waveforms of the memristor conductance and output neuron voltage changing with an increase in the number of clock cycles during the training time.

Figure 4a shows a cross-sectional view of the evaluated memristor device composed of a top electrode, memristor film, and bottom electrode [36,38]. The measured and modeled butterfly curves are also shown in Figure 4a [36]. Here, the black line with the solid box represents the experimentally measured butterfly curve. The red line denotes the modeled butterfly curve. The measurement of the butterfly curve of the memristor was performed with the Keithley 4200 semiconductor parameter analyzer (Semiconductor Characterization System, Tektronix, Inc., Beaverton, OR, USA) by using the probe station with a shield box. The mathematical modeling equations can be found in detail in the previous reference [36,39]. The modeling equations for the memristor were programmed in Verilog-a. The simulation performed with the programmed Verilog-a code using CADENCE SPECTRE circuit simulator (Cadence Design Systems, Inc., San Jose, CA, USA) is depicted in Figure 4a.

Figure 4b shows the waveforms of the simulated free-phase and nudge-phase circuits presented in Figure 2a–c. In the upper, the memristor conductance is simulated with an increasing number of clock cycles during the training time. Here, the solid line represents the change in the R1f conductance with respect to the number of cycles, and the dotted line denotes the conductance of the other R2f. As the training continues, the memristor conductance is updated according to the modified EP algorithm with the learning rule given by Equation (3) to reduce the loss between the target label and the EP circuit output. In Figure 4b, the EP circuit output voltages are shown in the lower graph. The solid line indicates the output neuron voltage of Y1f+-Y1f- with the increasing number of clock cycles. The dotted line indicates the other output neuron voltage of Y2f+-Y2f-. Both of them appear to become closer to their target labels. Here, the simulation in Figure 4b was performed in CADENCE SPECTRE. The Verilog-a model of the memristor has already been explained in Figure 4a. The detailed equations for modeling the transient characteristics of the memristor can be found in the previous reference [36].

In Figure 4b, the conductance of the simulated memristor appears saturated after 100 clock cycles. This is because the high-resistance state (HRS) and low-resistance state (LRS) of the simulated memristor are 1 MΩ and 10 kΩ, respectively. The HRS and LRS values are obtained from the measurements presented in Figure 4a. When the read voltage is 1 V, the measured memristor resistance is 1 MΩ and 10 kΩ by Ohm’s law for the HRS and LRS sates, respectively, as shown in Figure 4a. Once the memristor conductance reaches HRS or LRS, it cannot exceed HRS or LRS, although more programming pulses are applied to the device.

**Figure 5:**
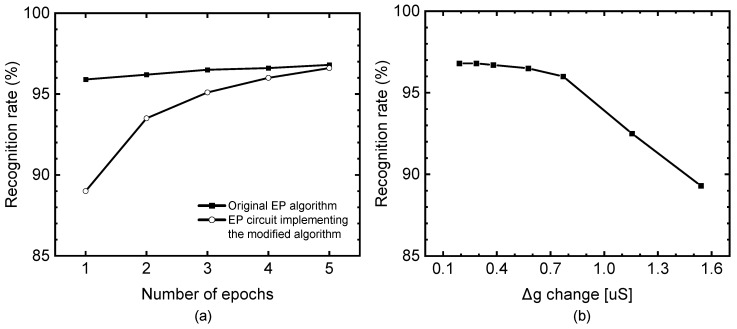
(**a**) Simulated MNSIT recognition rate for the original EP algorithm and the new EP circuit implementing the modified EP algorithm. The original EP algorithm uses the learning rule given by Equation (2) and the proposed EP circuit is trained by the learning rule given by Equation (3). (**b**) MNIST recognition rate simulated for the new EP circuit implementing the modified EP algorithm under varying ∆g change.

Figure 5a compares the recognition rates of the original EP algorithm and the EP circuit implementing the modified EP algorithm. The original and modified EP algorithms are presented in Figure 1a,b, respectively. Here, the dataset used in this simulation is the MNIST hand-written digits dataset [40]. In the MNIST dataset, the numbers of training and testing vectors are 60,000 and 10,000, respectively. The original EP algorithm was simulated with PyTorch, and the recognition rate was simulated as high as 96.9% when the number of epochs was 5. The modified EP algorithm shown in Figure 1b can be implemented by using the memristor–CMOS hybrid circuits, as shown in Figure 2 and Figure 3. The recognition rate of the EP circuit implemented with the memristor–CMOS hybrid circuit was simulated using Ngspice circuit simulator and Python. The results of the simulation revealed a recognition rate as high as 96.7% for epoch number = 5. The gap between the original EP algorithm and the EP circuit implementing the modified EP algorithm was as little as 0.2%. The gap of the recognition rate between the original EP algorithm and the proposed EP circuit could be caused by the difference in learning rule. As explained earlier, the original EP algorithm uses the learning rule given by Equation (2). By contrast, the proposed EP circuit implementing the modified EP algorithm is trained by the modified EP learning rule given by Equation (3). The amount of conductance update per clock cycle in Equation (2) can be changed according to the amount of output error. However, the amount of ∆g in Equation (3) is fixed regardless of the amount of output error. The fixed amount of ∆g can degrade the performance of the neural network, as shown in Figure 5a.

Figure 5b compares the recognition rate with varying ∆g change. Here, ‘∆g change’ means a fixed conductance change per programming pulse and can be calculated using the following equation:

(4) $$∆g=\frac{\left(gLRS-gHRS\right)}{2^{N}}$$

where gLRS and gHRS are the LRS and HRS conductances, respectively, and N denotes the number of bits. The LRS and HRS used in the simulation were 10 kΩ and 1 MΩ, respectively. For a memristor precision of 8 bits, the ∆g change was as small as 0.38 μS, and the recognition rate was as high as ~97%. Moreover, when the memristor precision was 7 bits, the ∆g change was as small as 0.77 μS; in this case, the recognition rate was 96%. From the experimental results published recently, many memristors have been reported to be able to have higher than 7-bit resolutions [41,42]. When the memristor precision becomes less than 7 bits, the recognition rate appears to start degrading considerably.

Another notable point here is the memristor variation problem. The fabrication process for memristors has not matured until now. The measured memristor conductance shows device-to-device, wafer-to-wafer, and lot-to-lot variations. To consider the variation effect, four cases of random variation are assumed in the memristor crossbar and CMOS hybrid circuits calculating the free-phase and nudge-phase solutions. Specifically, the conductance of each memristor shown in Figure 2a,b is assumed to have random variations as much as 0%, 1%, 3%, and 5%. A 0% variation means that there is no variation. The circuit simulation with random variations of 0%, 1%, 3%, and 5% was performed using Ngspice and Python. For the memristor conductance variation = 0%, the recognition rate was as high as 96.7%. If the conductance variation became 1%, the rate decreased to 95.4%. Further, when the conductance variation was 3% and 5%, the rate became 93.9% and 92.1%, respectively. Comparing the 0% and 5% variations, the rate loss was only 4.6%, indicating that the EP circuit in Figure 2a,b can calculate the weight update well despite a memristor conductance variation as large as 5%.

## 4. Conclusions

Analog circuits are proposed herein to realize the EP algorithm in practical and implementable hardware. This work has two major contributions for realizing the EP algorithm as practical analog circuits. First, the free-phase and nudge-phase solutions are calculated by the proposed analog circuits simultaneously, not at different times. Because of this process, the analog or digital memories with converting circuits between digital and analog domains for storing the free-phase solution temporally can be eliminated in the proposed EP circuit. Second, a simple EP learning rule relying on a fixed amount of conductance change per programming pulse is proposed and implemented in peripheral circuits. The modified EP learning rule could make the weight update circuit practical and implementable without using the complicated program verification scheme. The proposed memristor conductance update circuit was simulated and verified for training synaptic weights on memristor crossbars. The simulation results indicated that the proposed EP circuit is suitable for edge intelligence hardware for realizing physics-based on-device learning.

## Data Availability

Data available on request due to some restrictions of research project policy.
